# Resin-Dentin Bonding Interface: Mechanisms of Degradation and Strategies for Stabilization of the Hybrid Layer

**DOI:** 10.1155/2019/5268342

**Published:** 2019-02-03

**Authors:** D. E. Betancourt, P. A. Baldion, J. E. Castellanos

**Affiliations:** ^1^Universidad Nacional de Colombia, Facultad de Odontología, Departamento de Salud Oral, Colombia; ^2^Universidad Nacional de Colombia, Facultad de Odontología, Departamento de Ciencias Basicas, Colombia

## Abstract

Several studies have shown that the dentin-resin interface is unstable due to poor infiltration of resin monomers into the demineralized dentin matrix. This phenomenon is related to the incomplete infiltration of the adhesive system into the network of exposed collagen fibrils, mainly due to the difficulty of displacement and subsequent replacement of trapped water between interfibrillar spaces, avoiding adequate hybridization within the network of collagen fibrils. Thus, unprotected fibrils are exposed to undergo denaturation and are susceptible to cyclic fatigue rupture after being subjected to repetitive loads during function. The aqueous inclusions within the hybrid layer serve as a functional medium for the hydrolysis of the resin matrix, giving rise to the activity of esterases and collagenolytic enzymes, such as matrix metalloproteinases, which play a fundamental role in the degradation process of the hybrid layer. Achieving better interdiffusion of the adhesive system in the network of collagen fibrils and the substrate stability in the hybrid layer through different strategies are key events for the interfacial microstructure to adequately function. Hence, it is important to review the factors related to the mechanisms of degradation and stabilization of the hybrid layer to support the implementation of new materials and techniques in the future. The enzymatic degradation of collagen matrix, together with resin leaching, has led to seeking strategies that inhibit the endogenous proteases, cross-linking the denudated collagen fibrils and improving the adhesive penetration removing water from the interface. Some of dentin treatments have yielded promising results and require more research to be validated. A longer durability of adhesive restorations could resolve a variety of clinical problems, such as microleakage, recurrent caries, postoperative sensitivity, and restoration integrity.

## 1. Introduction

Composite resin is a restorative material widely used in dentistry for filling dental cavities and cementing indirect restorations and aesthetic restorations [1, 2]. The resin-dentin bond depends on the infiltration of the adhesive system into the collagen matrix of the dentin, which is exposed through acid conditioning. The resin-dentin interdiffusion zone, called the “hybrid layer,” fulfills a fundamental function in the micromechanical retention of the restoration [3]. It has been established that the infiltration of collagen by the adhesive is incomplete since its penetration capacity is lower than the depth of conditioning of the etching agent. Additionally, removing residual water in the dentin matrix is difficult [4, 5]. Both of these are reasons why a portion of collagen remains unprotected, which results in the activation of endogenous proteases, called extracellular matrix metalloproteinases (MMPs) and cysteine cathepsins (CTs), present in dentin. As collagenolytic enzymes, MMPs and CTs hydrolyze the organic matrix of demineralized dentin, an event that triggers hybrid layer degradation [6, 7].

To counteract the effect of MMPs, the use of nonspecific synthetic inhibitors, such as chlorhexidine (CHX) [8, 9], has been suggested. However, it has been shown to be effective only in short and medium terms due its binding to dentin being electrostatic in nature [10]. As a result, its inhibitory capacity decreases in less than 2 years [11]. On the other hand, Breschi et al. [12] reported the effectiveness of 0,2% CHX to inhibit MMPs activity in acid-etched adhesive-infiltrated dentin aged for 2 years. Recently, the experimental use of cross-linking agents of collagen has been proposed as a strategy. This technique promotes resistance to enzymatic degradation and has the ability to inhibit the activity of MMPs. Flavonoid-type polyphenolic compounds, such as proanthocyanidins, quercetin, and curcumin, have chemical structures that favor their function as cross-linking agents. However, their benefits in clinically relevant protocols have not been shown since the application times required and their depth of penetration do not make them effective within clinical protocols [13–15].

This hydrolytic degradation of the adhesive interface generates adverse clinical consequences, such as dentin hypersensitivity, marginal pigmentation, and possible secondary caries, thus decreasing the longevity of the restorations [16]. Such events result in high biological and economic costs associated with the need to replace restorations [4].

The objective of this study was to review the literature to identify factors that influence degradation of the resin-dentin adhesive interface and strategies that have been proposed to stabilize the hybrid layer and improve the durability of adhesive restorations.

## 2. Methods

An electronic search was carried out to identify relevant manuscripts in the following databases: PubMed, Scielo, Cochrane, Elsevier, EBSCO, LILACS, and Web of Science, using terms selected according to the Medical Subject Headings (MeSH): bonding, collagen, cross-linking reagents, matrix metalloproteinases, dentin, dentin bonding agents, endopeptidases, cysteine cathepsins. The reference lists of the included articles were also reviewed. There was no restriction of year or language for the publications, and the last search was conducted in Nov 2018. This review aimed to collect the most outstanding information that described the mechanisms that occur during degradation processes of resin adhesives and collagen matrix, as well as some of the experimental strategies to stabilize the resin-dentin interface, mainly related to inhibition of MMPs and collagen cross-linking.

### 2.1. Dentin as a Substrate for Adhesion

Dentin, a biological, complex, and dynamic tissue, underlies the dental enamel and is related histologically, embryologically, and functionally to the dental pulp. Odontoblasts are highly specialized cells that produce both collagen and noncollagen proteins to build the dentinal extracellular matrix [17]. Dentin is composed of 47% (vol) apatite crystals, 20% (vol) water, and 33% (vol) organic material. Dentin has a tubular structure in a radial arrangement, where tubules run from the pulp to the dentinoenamel junction (DEJ) surrounded by intertubular dentin. Close to the pulp, the tubule number is 45,000-65,000/mm^2^ and reaches 22% of the dentinal area, while in outer dentin it is 15,000-20,000/mm^2^ representing 1% of the dentinal area. In the same way, the diameter of the tubules close to the pulp is larger (3-4 *μ*m) and smaller near the DEJ (1,7 *μ*m) [18]. The regional differences in the intertubular area and tubule orientation may influence the efficacy of the dentinal adhesives [19].

Type I collagen makes up 90% of the organic material, with noncollagenous proteins accounting for the remainder [18, 20]. These proportions vary according to the region of the tooth and are affected by physiological processes, such as aging, and pathological processes, such as dental caries [21]. Dentin conditioning by acid etching also alters the composition of dentin, whereby the amount of water present changes from 18% to 50-70% by volume, with great implications on the physical characteristics of the tissue [22].

Collagen is a protein composed of a sequence of amino acids: glycine, lysine, proline, and their hydroxylated products, mainly hydroxyproline and hydroxylysine. It is synthesized from a larger molecule, tropocollagen, which is cleaved at the level of terminal carboxyl and amino groups with a periodicity of 67-69 nm, which in turn facilitates cross-linking. The intrinsic cross-linking ability of collagen is enhanced by enzymatic and nonenzymatic reactions. Enzymatic reactions form lysine-lysine covalent bonds, which are mediated by the hydroxylation of lysine, glycosylation, and molecular turnover rate. Nonenzymatic cross-linking involves oxidation and glycation processes [23, 24].

**Figure 1:**
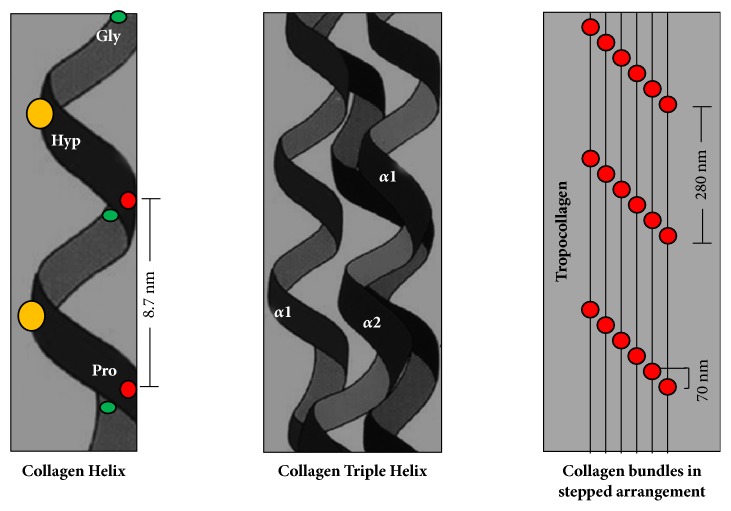
**Structure of collagen**. Each collagen helix is called the *α* chain, which is a levorotatory strand with about 3 residues per turn of glycine (Gly), proline (Pro), and hydroxyproline (Hyp) sequences. The quaternary structure of the collagen fibril is formed from the supercoiling of three *α* chains to form a triple dextrorotatory helix.

The quaternary structure of collagen has a triple helix with an arrangement that makes it very stable and resistant to degradation. Collagen plays a prominent role in the tensile strength, the elastic modulus and biochemical properties of dentin, which depend on its degree of cross-linking [25] (Figure 1).

**Figure 2:**
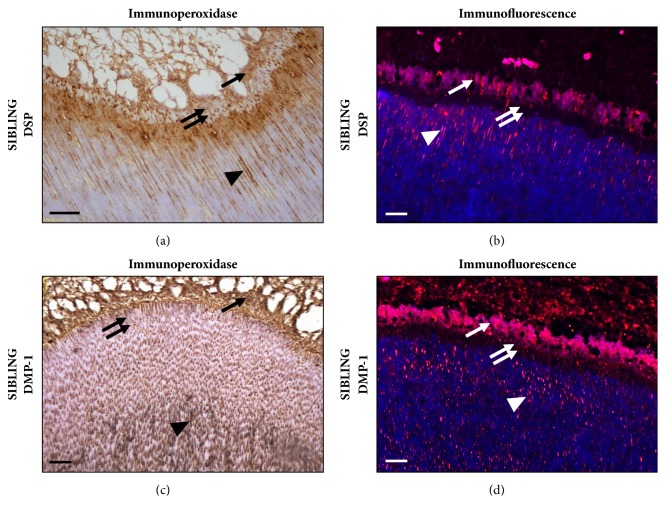
**Location of SIBLING proteins in mineralized dentin.** An immunocytochemistry analysis using one of two primary antibodies, polyclonal antidentin sialophosphoprotein (Abcam, Cambridge, MA, USA) which is specific to dentin sialoprotein (DSP) or polyclonal antidentinal matrix protein (DMP)-1 (Sigma-Aldrich, St Louis, MO, USA) and visualized using peroxidase (brown color in (a) and (c)) or Alexa Fluor 594 (red color in (b) and (d)) coupled streptavidin under fluorescence microscopy revealed the localization patterns of DSP and DMP-1. DSP and DMP-1 are strongly expressed in the odontoblastic layer (arrow) and with less intensity in the predentin area (double arrow) and in the dentin mineralization front (head arrow). (a) and (b) immunopositive staining for DSP in the mineralized dentin. (c) and (d) immunolocalization of DMP-1. These non-collagenous proteins interact with collagen fibrils and control initiation and growth of apatite crystals on dentinal matrix. Mineralized dentin counterstained with hematoxylin (a and c) and Hoechst blue 33342 (b and d). Bar corresponds to 100 *μ*m. Courtesy of Baldion et al. [29].

Noncollagenous proteins, such as proteoglycans (PG) (e.g., chondroitin-4/6-sulfate, decorin, biglycan, lumican, and fibromodulin) [26, 27], and small integrin-binding ligand N-linked glycoproteins (SIBLING), such as bone sialoprotein, osteopontin, protein dentinal-1 matrix, and dentin sialophosphoprotein, play important roles in dentinogenesis, including regulation functions and control of crystal growth, fibrillogenesis, and mineralization [28] (Figure 2).

### 2.2. MMPs: Structure and Function

**Figure 3:**
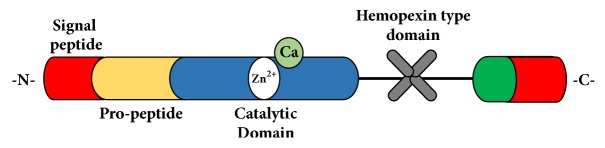
**Basic structure of the MMP.** MMPs consist of four main domains: a signal peptide that directs the secretion of the protein to the outside of the cell, a propeptide region that keeps the enzyme inactive until it is proteolytically cleaved, a catalytic domain that contains zinc and calcium and to which the cysteine of the propeptide region binds to keep it inactive (cysteine switch), and a hemopexin-like domain that mediates substrate specificity and interactions with endogenous inhibitors. MMP-2 and MMP-9 possess a fibronectin-type domain for matrix binding.

MMPs are a family of enzymes and 23 types have been described in humans. They are dependent on calcium and zinc for activity and are characterized by having four domains in their composition: the peptide-signal domain, the propeptide domain (composed of cysteine with its sulfhydryl groups), a catalytic domain (with zinc at the site of catalytic activity together with histidine and calcium residues), and a hemopexin-type domain that serves to bind to the substrate and which is the site where specific tissue inhibitors bind [30] (Figure 3).

These enzymes are produced by leukocytes and fibroblast-like cells capable of synthesizing extracellular mineralized matrix, including the cells of the dental pulp. They are secreted in the form of proenzyme (inactive enzyme), remaining as zymogens until activated. The proenzyme is characterized by binding to the sulfhydryl groups present in the cysteine component of the propeptide domain, with zinc, although present in the catalytic domain, being unavailable for catalytic activity. The process of activation of the enzyme is initiated by the breakdown of the zinc-cysteine interaction, which is called the cysteine switch. This process is key to understanding the functioning of these enzymes and their possible inhibition [31–34].

During tooth development, close ectomesenchymal communication is required, and MMPs play an important role in this interaction. In early stages of odontogenesis, this interaction determines dental morphogenesis and in late stages, determines the differentiation of odontoblasts and ameloblasts [35, 36]. Mesenchymal cells express, at least, MMP-1, MMP-2, MMP-3, MMP-8, MMP-9, MMP-14, and MMP-20. Additionally, the role of MMPs in the process of reabsorption of extracellular matrix proteins has been proposed as a regulatory mechanism necessary for correct mineralization of the dental structure [37]. Some in the form of proenzymes are trapped inside the mineralized dentin matrix during dentinal development [38], and because of their collagenolytic protease activity, they can hydrolyze the components of the extracellular matrix when activated by physical or chemical stimuli [39]. These proteases play a central role in several physiological processes, such as development, tissue remodeling, and angiogenesis [40].

MMPs are involved in different pathological processes, such as periodontal disease and dental caries. Recent studies have revealed their role in the breakdown of the collagen matrix in the pathogenesis of caries [33, 41], with potential and relevant implications for dentin binding [42]. In addition, they may be present in saliva [43], peritubular dentin, and presumably, dentinal fluid [44].

Of the proposed classifications to name MMPs, the one most used was established based on the substrate on which they have activity. Therefore, they are named as collagenases (MMP-1, MMP-8, MMP-13, and MMP-18), gelatinases (MMP-2 and MMP-9), stromelysins (MMP-3 and MMP-10), matrilysins (MMP-7 and MMP-26), and membrane-type MMP (MMP-14, MMP-15, MMP-16, and MMP-24) (Bali et al., 2016). MMPs found in human dentin are MMP-2, MMP-9 [40], MMP-8 [32], MMP-3 [45] and MMP-20 [46].

### 2.3. Degradation of the Adhesive Interface

Composite resins, as restorative materials, base their retention on an adhesive process that unites them to the dental structure. Adhesion to dentin is a clinical challenge, because it is a tissue of heterogeneous composition, with high organic content and the presence of moisture [2, 22]. The adhesion of resinous systems to the dentin requires the infiltration of collagen fibrils that have been exposed by previous acid conditioning, with the resin monomers present in the adhesive generating a resin-dentin interdiffusion zone, called the hybrid layer [3]. This is necessary for the micromechanical retention of the restoration.

It is widely accepted that after the use of current dental adhesives, a degradation of the dentin-resin adhesive interface occurs [4]. Among the factors that intervene with the degradation of the adhesive interface, the following have been proposed:

#### 2.3.1. Degradation of the Adhesive Resin

The use of hydrophilic monomers in adhesive systems, such as 2-hydroxyethyl methacrylate (HEMA), seeks to improve infiltration of the exposed collagen network, which is inherently humid. This has been reported to result in an immediate improvement of bond strength [47]; but the longevity of this dentin-resin bond is compromised with the use of these adhesive systems [48–50].

The presence of water in the adhesive interface generates a weak hybrid layer, in which the phenomena of hydrolysis and leaching of resin adhesives occur [16, 51]. Current adhesives include in their formulation hydrophilic and hydrophobic components that, in aqueous solution, produce nanophase separation of adhesives [52]. The hydrophilic elements penetrate the interior of the hybrid layer, while the hydrophobic monomers remain on the surface. Being hydrophobic, the camphorquinones do not penetrate, leading to inadequate polymerization in the deepest zone of the hybrid layer [53].

**Figure 4:**
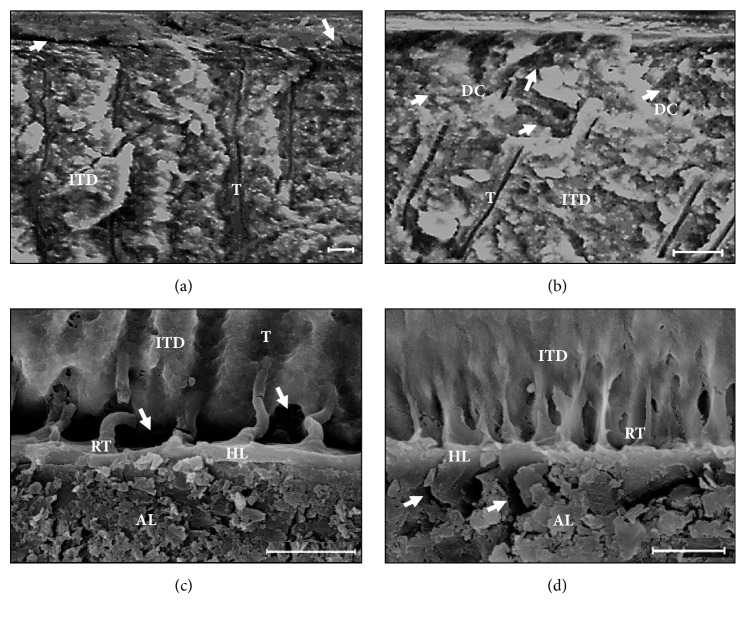
**Resin-dentin interface with 150 days of aging by SEM.** (a) Arrows show voids in the deepest portion of hybrid layer. (b) Degradation of collagen fibrils is evidenced (arrows) in the adhesive interface. (c) Loss of collagen in the intertubular dentin around the resin tags (arrows). (d) Degradation of the bonding interface with formation of water channels (arrows) and hydrolytic degradation of the resin adhesive. Bonded interfaces were created with Adper Single Bond 2 (3M ESPE, St. Paul, MN, USA). (SEM) scanning electron microscopy, (ITD) intertubular dentin, (T) dentin tubule, (DC) degraded collagen, (RT) resin tags, (HL) hybrid layer, (AL) adhesive layer. Bar corresponding to 10*µ*m. Courtesy of Betancourt DE and Baldion PA with permission.

The incomplete polymerization of the hydrophilic portion of the adhesives and the aqueous sorption of the material allow the mobilization of water, which forms large aqueous channels within the hybrid layer [54] (Figure 4.). Additionally, the action of esterase enzymes that come from saliva, pulp, and bacteria [55] break the ester bonds present in the HEMA, which generates hydrophilic cytotoxic by-products such as ethylene glycol, as well as hydrophobic ones such as methacrylic acid [56].

This phenomenon can be triggered not only with etch-and-rinse (E&R) adhesives but also with self-etching (SE) adhesives [31, 57]. Several studies, such as those by Nishitani et al. [7] and Mazonni et al. [5], have established that the monomeric systems incorporated in self-etching adhesives are also susceptible to hydrolytic degradation and allow collagenolytic and gelatinolytic activities within the collagen matrix due to their low pH.

#### 2.3.2. Incomplete Infiltration of the Adhesive in the Exposed Collagen Network

**Figure 5:**
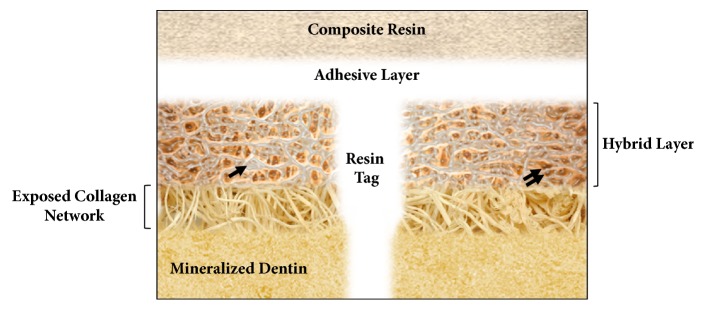
**Schematic illustration of the instability of the hybrid layer**. Apatite crystals are removed from dental hard tissues with acid conditioning and should be replaced by resin monomers. Note the incomplete infiltration of the adhesive in the collagen matrix that remains with interfibrillar spaces saturated with water (arrow). In addition, the possibility of discrepancy between the depth of infiltration of the adhesive and that of conditioned dentin leaving collagen exposed without hybridizing. The resin tags partially seal the dentinal tubules and decrease the permeability of the dentin. The resin monomers do not penetrate homogenously into the collagenous network (double arrow) [58] (with permission).

In the E&R technique, the difference between penetration of the adhesive and action of the conditioning acid agent leads to an incomplete hybridization of the exposed collagen network. As such, a portion of the collagen fibrils remain uninfused, being more susceptible to hydrolytic degradation, which leads to nanopercolation [58] (Figure 5).

The inability of resin monomers to replace both free and collagen-bound water present in the inter- and intrafibrillar compartments is a limitation to achieve a complete and stable hybrid layer [59, 60].

Additionally, highly hydrated proteoglycan hydrogels are found in interfibrillary spaces [61]. These act as filters that trap the monomers of large molecules, such as BisGMA, and only allow the passage of small monomers, such as HEMA, toward the base of the hybrid layer. HEMA produces weak linear chains that, when subjected to stresses, lead to failure due to cyclic fatigue of the collagen chains [62].

#### 2.3.3. Degradation of Collagen by Endopeptidases

Pashley et al. [6] were the first to demonstrate the degradation of collagen fibrils in the absence of bacteria, suggesting proteolytic activity within the dentin. Dentin contains a large amount of MMPs in inactive form, which are trapped in the tissue, from the mineralization process and can be activated by different chemical and physical mechanisms. The incomplete infiltration of the collagen network by resin monomers makes it especially vulnerable to hydrolytic degradation mainly by MMPs [14]. These enzymes have a great capacity to degrade almost all components of the extracellular matrix, acting in a synchronous manner, so that some MMPs require previous activation of other proteases. This is how, for example, MMP-8 has the ability to degrade collagen, resulting in the release of 3/4- to 1/4- length peptides, denaturing the triple-helical structure, allowing the subsequent activity of gelatinases (MMP-2 and MMP-9) to digest peptide residues [34].

MMPs can be activated in vivo by proteases and other MMPs, in vitro by chemical agents such as modifiers of the sulfhydryl groups, chaotropic agents, and reactive oxygen, as well as physical agents such as low pH and heat [30, 34, 39].

The degradation of collagen fibrils in vivo has been directly correlated with the activation of MMPs induced by the application of dentinal adhesive systems [11, 63, 64]. Initially, the acid conditioning procedure was believed to favor the release and activation of proenzymes trapped inside the mineralized dentin [5], which induces a collagenolytic activity within the hybridized dentin. However, some authors have reported that the very low pH (0.7-1) of phosphoric acid in the conditioning can denature MMPs by preventing their action [5–7]. In contrast, when mixing powder of demineralized dentin with SE adhesives it has been shown that the pH, initially very low, rises to neutrality quickly, and the SEM revealed the presence of a dense insoluble precipitate that is deposited on the surface of the conditioned collagen fibrils, which could temporarily prevent the activity of MMPs [65]. Sabatini & Pashley [66] propose that acid phosphate groups bind with Ca^2+^ and become deposited on the surface of the dentin.

The action of MMP-2 and MMP-9 has been confirmed by means of specific tests for different types of dentinal adhesives. Lehmann et al. [57] reported that the application of the adhesive increases the synthesis of MMP-2 in human odontoblasts, which possibly increases its action by migration through the dentinal tubules to the hybrid layer. With regard to SE adhesives, Mazzoni et al. [5] reported that there is evidence that partially demineralized dentin activity was found in MMP-2 and MMP-9 in human dentin. This effect is attributed to the low pH which triggers the cysteine switch and activates latent forms of the enzymes to exert their effect on the catalytic domain. It also decreases the inhibitory activity of tissue inhibitors of MMP.

CTs are host-derived proteases present in dentin and play an important role in the matrix collagen degradation. There are 11 CTs in humans [67]. In dentin, has been described the CT-K (the most potent collagenase in mammalian), CT-L and CT-B [68]. CTs form collagen active complexes with PG glycosaminoglycan (GAG) side chains, which can degrade collagen at multiple sites and generate multiple collagen fragments [67]. It has been suggested there is synergistic activity between MMPs and CTs, as they are located very close together and near to the target substrate. The acidic activation of CTs may further activate dentin-bounded MMPs [69].

### 2.4. Strategies for the Stabilization of the Adhesive Interface

#### 2.4.1. Nonselective MMPs Inhibitors

Within this group, the most commonly used substance is CHX, which efficiently inhibits the activity of MMPs for a short time even at low concentrations such as 0,2% [70, 71]. The effect of CHX is maintained for 18 months, at which time, the degradation of the adhesive interface begins. Although very little data have been published about the mechanism of action of CHX, this may be because chlorine behaves like an amphiphilic molecule that can bind to zinc of the catalytic domain of MMP, preventing its hydrolytic activity. Additionally, CHX is a cationic molecule that binds to the anionic molecules of both mineralized and demineralized dentin [71]. The union of CHX with dentin is of the reversible electrostatic type and the activity time depends on the substantivity of CHX to treated dentin [9, 14, 71].

#### 2.4.2. Biomodification of Dentin

Advances in tissue engineering have limited application in dental hard tissues due to their poor capacity to regenerate. This has led to the search for different strategies in which biomodification improves the physical properties of dentin by modifying their biochemistry.

(*1) Physical Agents.* The use of photo-oxidative techniques by the action of ultraviolet (UV) light requires the presence of an oxygen singlet, which is the most reactive and unstable type of the dioxygen molecule. Vitamin B2 (riboflavin) has been shown to be an important source of oxygen free radicals, including oxygen singlets. This vitamin, when activated by UV light, can induce the formation of covalent bonds mediated by oxygen singlets, between the amino group of glycine of a collagen chain, and the carbonyl groups of proline and hydroxyproline of side chains, leading to a cross-linking effect of collagen [24, 72, 73].

(*2) Nonspecific Synthetic Agents of Collagen Cross-Linking.* Cross-linking agents are substances that can bind to amino and carbonyl groups of the amino acid residues of the collagen, which stabilizes its structure and encompass it to make it more resistant to enzymatic degradation. Glutaraldehyde (GA) has great affinity for terminal amino groups, mainly the ɛ-amino groups of peptidyl-lysine and hydroxylysine residues of collagen [74]. Less cytotoxic, carbodiimide (EDC) requires activation of the carboxylic groups of glutamic and aspartic acids to form an O-acyl isourea intermediate, which then reacts with the amino groups of lysine and hydroxylysine to form amino cross-linkages with collagen, with the release of urea. It has been reported that EDC requires a relatively long time (1 h) to cross-link the collagen, which limits its clinical use [75].

Although these substances have been shown to be effective in providing better stability of the adhesive interface [76], their biocompatibility has been questioned [24, 77]. Scheffel et al. (2015) [78] evaluated the cytotoxic effect of 5% GA and different concentrations of EDC on odontoblast-like cells with dentin barriers, and they concluded that 0,1; 0,3 and 0,5 M EDC and 5% GA did not induce transdentinal cytotoxic effect on odontoblast-like cells.

(*3) Cross-Linking Agents of Natural Origin.* These are antioxidant substances capable of promoting cross-linking with collagen and can inhibit the activity of MMPs. The cross-linking of the bioactive substances with collagen is nonenzymatic. Type I collagen provides tensile strength and cohesiveness properties [79] by endogenous covalent intermolecular cross-linking. The elastic strength-strain curve increases with an increasing degree of cross-linking [80].

The demineralized dentin has a significant decrease in its mechanical properties [81] and dentin biomodification improves mechanical properties through nonenzymatic collagen cross-linking [25], which can be attributed to the degree of cross-linking of the denuded collagen matrix.

It has been proposed that cross-linkers can inhibit the protease activity of MMPs and CTs by different mechanisms, such as down regulation of endogenous protease expression, protease inactivation/silencing and protection of cleavage sites within collagen modifying and hiding cleavage sites in the substrate [82], and avoiding the oxidation of cysteine switch. Within this group, anthocyanidins have been studied, mainly oligomeric proanthocyanidin. This is derived from grape seeds and improves the mechanical properties of previously demineralized dentin, such as tenacity and elastic modulus [83]. Additionally, an increase in the contact angle of the water and a decrease in water vapor permeability has been demonstrated [84]. However, the effects of these substances require very long application times (10 min to 1 h), a situation that does not make them clinically applicable [13]. Furthermore, a reduction in the degree of conversion has been reported by inhibiting the polymerization of resin monomers [84] and brown pigmentation in dentin [85].

(*4) Biomimetic Remineralization.* The use of amorphous calcium phosphate nanoprecursors seeks to reproduce the natural biomineralization mechanisms of dentin [86, 87]. Although the incorporation of crystals in demineralized dentin has been achieved in vitro by means of lateral diffusion mechanisms, this process does not yet seem to be useful clinically [88].

#### 2.4.3. Removal of Residual Water Not Bound to Collagen in the Hybrid Layer

Most methacrylates are hydrophobic and insoluble in water, which is why many manufacturers use ethanol as a solvent to ensure that they remain in solution in one phase. The application of these adhesives in water-saturated dentin after acid conditioning, generates separation in nanophases of adhesives [52]. Pashley et al. [89] propose a change in the technique of wet adhesion where residual water is replaced by ethanol, which achieves a better penetration of the resin monomers in the collagen network and avoids the separation in nanophases of the adhesive, but they have an effect on partial inhibition of MMPs, less than alcohols with 4 methylene groups, which inhibit MMPs more effectively [90]. The water molecule can be found attached to the collagen in a three-layer arrangement, or it can be found free. Ethanol can remove free water and half of the third layer of bound water [60], which decreases the separation between the collagen matrix and resin monomers and in turn, the possibility of action of collagenolytic enzymes.

Another approach to remove residual water is the use of SE primer adhesives with higher concentrations of functional molecules, such as 10-MDP (20-25 vol%), to which has been added only the water necessary to ionize the acidic monomers (20–25 vol%), which decreases the amount of water in the demineralization front [91]. This dry technique proposes use of SE primer adhesives for 10 s, the conditioned dentin is dried and then sealed with a solvent-free adhesive, which produces a thin hybrid layer (1 *μ*m) with good durability, but still prone to loss of bond strength over time [92, 93].

## 3. Conclusions

Dentin bonding is a challenge in clinical practice because it is a heterogeneous substrate with high protein content and is inherently humid. The presence of water in the adhesive interface constitutes a vehicle for the hydrolytic degradation of components of the hybrid layer. The stability of the dentin-resin interface is a necessary requirement for the durability of restorations; thus, different strategies have been proposed to control the different factors that result in its degradation.

Wet bonding techniques improve the immediate adhesive strength, and the use of dentin treatments with saturated alcohols, such as 100% ethanol, promotes the removal of water from the exposed collagen network to facilitate the penetration of adhesive monomers. However, this protocol requires a lot of clinical time and the durability of the restoration is compromised by not preventing the interface degradation.

Although CHX has been shown to have a beneficial effect on inhibiting the activity of MMPs in the adhesive interface, the duration of its effect for a limited time does not constitute a final solution to the problem of the degradation of the hybrid layer.

The biomodification of dentin is presented as an option that shows promising results. Cross-linking of the collagen in demineralized dentin improves the physical properties of dentin and makes it more resistant to degradation by the action of collagenolytic enzymes, in addition to the ability to inhibit the activity of MMPs and CTs by some cross-linking agents. However, all studies have been conducted in vitro since these substances have limitations that do not allow them to be used in a relevant clinical protocol. Due to this unresolved problem, it is necessary to continue with the search for new substances and techniques that can be used in a clinical protocol to improve the stability of the resin-dentin bonding interface, with which more durable adhesive restorations can be obtained.
